# Mesenchymal Stem Cell-Derived Extracellular Vesicles: A Novel Cell-Free Therapy for Sepsis

**DOI:** 10.3389/fimmu.2020.00647

**Published:** 2020-04-21

**Authors:** Yanwei Cheng, Xue Cao, Lijie Qin

**Affiliations:** ^1^Department of Emergency, Henan Provincial People's Hospital, People's Hospital of Zhengzhou University, People's Hospital of Henan University, Zhengzhou, China; ^2^Department of Rheumatology and Immunology, Henan Provincial People's Hospital, People's Hospital of Zhengzhou University, People's Hospital of Henan University, Zhengzhou, China

**Keywords:** mesenchymal stem cell-derived extracellular vesicles (MSC-EVs), sepsis-induced acute lung injury, sepsis-induced acute kidney injury, sepsis-associated cardiovascular disorder, sepsis-induced liver injury

## Abstract

Sepsis remains a serious and life-threatening disease with high morbidity and mortality. Due to the unique immunomodulatory, anti-inflammatory, anti-apoptotic, anti-microbial, anti-oxidative, and reparative properties, mesenchymal stem cells (MSCs) have been extensively used in preclinical and clinical trials for diverse diseases and have shown great therapeutic potential in sepsis. However, concerns remain regarding whether MSCs can become tumorigenic or have other side effects. Extracellular vesicles (EVs) are a heterogeneous group of membrane-enclosed particles released from almost any cell and perform an important role in intercellular communication. Recently, it has emerged that EVs derived from MSCs (MSC-EVs) appear to exert a therapeutic benefit similar to MSCs in protecting against sepsis-induced organ dysfunction by delivering a cargo that includes RNAs and proteins to target cells. More importantly, compared to their parent cells, MSC-EVs have a superior safety profile, can be safely stored without losing function, and possess other advantages. Hence, MSC-EVs may be used as a novel alternative to MSC-based therapy in sepsis. Here, we summarize the properties and applications of MSC-EVs in sepsis.

## Introduction

In the recent “sepsis-3.0” consensus (1), sepsis is defined as a life-threatening, multiorgan dysfunction caused by a dysregulated host response to infection. In view of the high morbidity and mortality, sepsis has been described as the quintessential medical disorder of the 21st century. Despite continuous progress in the development of therapeutic strategies, sepsis is still a major clinical problem and remains the leading cause of death (2, 3) in the critically ill patient population, due to uncontrolled inflammation together with immunosuppression. In such a context, an investigation into novel therapies to ameliorate sepsis would be urgently needed.

With the emergence of stem cells as potential therapeutic agents for diverse diseases, attempts to use stem cells, especially mesenchymal stem cells (MSCs), to ameliorate sepsis in animal models have increased exponentially. Growing preclinical data have suggested that MSCs can directly modify pathophysiology and underlying injury mechanisms in sepsis through the immunomodulatory, anti-bacterial, anti-inflammatory, anti-oxidative, anti-apoptotic, and reparative properties. It is worth noting that MSCs have also been confirmed as having a therapeutic effect on sepsis in two clinical trials (4, 5). Even then, the utilization of MSCs as therapeutic agents for sepsis has raised several concerns over the past decade, including senescence-induced genetic instability, the heterogeneity of MSCs populations, quality assurance challenges of MSCs, and finding optimal MSCs tissue sources. Most crucially, the significant hurdles that potential therapeutics face in the future are MSCs safety concerns. It has been reported that MSCs play a dual role in immune regulation; on the one hand, MSCs exhibit immunosuppressive function (6), while on the other hand, MSCs may act as antigen-presenting cells, inducing an immune response (7). In addition, MSCs can potentially become tumorigenic either by direct malignant transformation of MSCs or indirectly by facilitating the growth of other tumor cells (8). So far MSCs have been found in several tumor types, including gastric adenocarcinoma (9), lipoma (10), and osteosarcoma (11), strongly indicating their involvement in tumor development. In light of this fact, the probability of the risks must be cautiously weighed against the potential benefits to every patient.

Recently, special attention has been paid to the extracellular vesicles (EVs) derived from MSCs (MSCs-EVs), which can function as shuttles for the delivery of a cargo that includes RNAs and proteins from parental to target cells and are as biologically active as the parent cells themselves (12–15). Hence, it is conceivable that the protective effect of MSCs on sepsis is at least partly due to the release of EVs.

## Characterization of MSC-EVs (Figure 1)

EVs are a heterogeneous group of membrane-enclosed particles that can be released from almost any cell. Based on the current knowledge of their size and biogenesis, EVs can be classified into three broad groups: exosomes (Exos), microvesicles (MVs), and apoptotic bodies. Exos (40–150 nm) bud from the endosomal system, whereas MVs (100–1,000 nm) are shed directly from the plasma membrane, and apoptotic bodies (1,000–5,000 nm) originate from apoptotic cells (16–18). In general, apoptotic bodies can be removed by the commonly used isolation method of ultracentrifugation (19). It should be noted that each individual cell can release both Exos and MVs simultaneously. However, so far it is impossible to unanimously distinguish MVs and Exos. In addition, most studies have not clearly defined the origin of EVs under study. Therefore, we here use EVs as an umbrella term to include both MVs and Exos.

**Figure 1:**
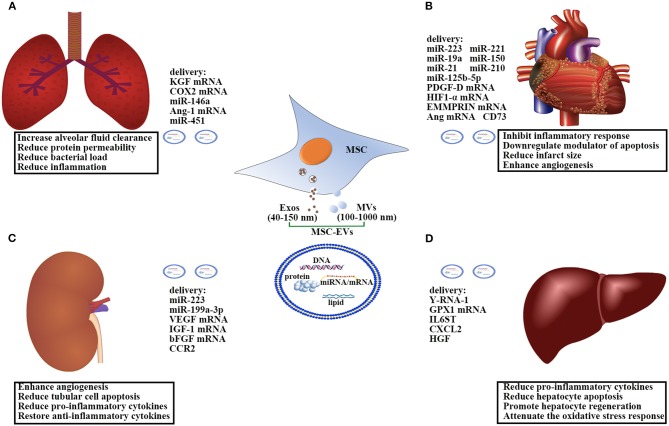
Therapeutic effects and mechanisms of the action of MSC-EVs in: **(A)** sepsis-induced acute lung injury; **(B)** sepsis-induced acute kidney injury; **(C)** sepsis-associated cardiovascular disorder; **(D)** sepsis-induced liver injury. EVs comprise a heterogeneous population of membrane vesicles of various origins. Here we use EVs as an umbrella term to include both MVs and Exos. MSC-EVs can shuttle various cargoes, including proteins, lipids, and nucleic acids, to the recipient cells to protect against sepsis-induced organ dysfunction. Ang, Angiopoietin; bFGF, Basic fibroblast growth factor; COX2, cyclooxygenase 2; CCR2, C-C motif chemokine receptor-2; CXCL2, Chemokine Ligand 2; EVs, Extracellular vesicles; Exos, Exosomes; EMMPRIN, Extracellular matrix metalloproteinase inducer; GPX1, Glutathione peroxidase 1; HIF1-α, Hypoxia-inducible factor 1-alpha; HGF, Hepatocyte growth factor; IL6ST, Interleukin 6 signal transducer; IGF-1, Insulin-like growth factor 1; KGF, Keratinocyte growth factor; MVs, Microvesicles; MSCs, Mesenchymal Stem Cells; miR, microRNA; PDGF-D, Platelet-derived growth factor D; VEGF, Vascular endothelial growth factor.

MSC-EVs are released from resting MSCs and can be markedly increased during cellular activation or cell stress (12, 20, 21). They contain a trove of bioactive substances such as proteins, lipids, and most importantly, nucleic acids, thereby playing an important role in immune modulation, pro-angiogenesis, anti-apoptosis, and tissue regeneration. Up to now, over 5,000 species of MSC-EVs proteins have been characterized, including mediators controlling self-renewal, differentiation, signal transduction, and additional MSCs antigens affecting the migration of MSCs (19, 22). In addition, MSC-EVs contain a number of adhesion molecules such as CD44, CD29, alpha 4-integrin, and alpha 5-integrin etc., which contribute to the identification of MSC-EVs (22). Accumulating evidence has demonstrated that MSC-EVs modulate immune response by producing cytokines, growth factors, and tolerogenic molecules, such as IL-10, IL-6, IL-37, and lipocalin-2 and transforming growth factor (TGF)-β, programmed death ligand-1(PD-L1), galectin-1, etc. (20, 23, 24). Other than proteins, MSC-EVs are enriched with nucleic acids, including mRNA, microRNA (miRNA), and DNA (25). It has been demonstrated that the transfer of miRNAs from MSC-EVs to target cells may be the underlying mechanism in alleviating injury of the kidney (26, 27), heart (28), liver (29), and brain (30). In recent years, more than 150 miRNAs have been identified in the cargo of MSC-EVs. These miRNAs are usually related to apoptosis, tumorigenesis, immune responses, angiogenesis, and organism development, such as miR-221, miR-23b, miR-125b, miR-451, miR-31, miR-24, miR-214, miR-122, miR-16, miR-150, and miR133b, etc. (19, 31–34). All the internal components in MSC-EVs are surrounded by a bilayer of lipids that play an important role in protecting them. Compared to their parent cells, EVs are enriched with some specific lipids (cholesterol, glycosphingolipids, and phosphatidylserine) (35–38), making them more functional. Our previous study (21) also showed that sphingosine-1-phosphate enriched within MSC-EVs could enhance the repair effect of MSCs for articular cartilage.

## Therapeutic Applications of MSC-EVs in Sepsis

Through delivering proteins and nucleic acids, MSC-EVs seem to hold many functions of the MSCs themselves and are described as a new mechanism of intercellular communication (39). Nowadays, increasing studies have clearly confirmed that EVs alone are responsible for the therapeutic effects of MSCs in sepsis-induced organ dysfunction, including sepsis-induced acute lung injury, sepsis-induced acute kidney injury, sepsis-associated cardiovascular disorder, and sepsis-induced liver injury (40). Accordingly, MSCs-EVs may be an alternative therapy to MSCs and will be the next-generation therapeutic route for sepsis.

### MSC-EVs and Sepsis-Induced Actue Lung Injury

Acute lung injury (ALI) is the most common organ injury in septic patients and leads to a greater mortality. In the past few years, the beneficial effects of MSCs on sepsis-induced ALI have been shown to be attributed to the release of EVs. Using LPS-induced ALI in an *ex vivo* perfused human lung, Park et al. (41) reported that MSC-EVs significantly increased alveolar fluid clearance and reduced protein permeability and numerically lowered the bacterial load in the injured alveolus. In a mouse model of *E. coli* endotoxin-induced ALI, Zhu et al. (42) also confirmed the similar beneficial effects of human MSC-EVs and further demonstrated that the protection was in part due to the delivery of keratinocyte growth factor (KGF) mRNA from MSC-EVs to the injured alveolar epithelium and lung endothelium. This falls in good accordance with Monsel's study (43) that MSC-EVs improved survival in ALI from *E. coli* pneumonia via a mechanism partially dependent on KGF secretion. The authors (43) further discovered that MSC-EVs could enhance monocyte phagocytosis of bacteria through transferring cyclooxygenase 2 (COX2) mRNA to monocytes, with a resultant increase in prostaglandin E2 (PGE2) secretion. PGE2 is essential for transforming the polarization of monocytes-macrophages M1 into M2 phenotype (44–46). In addition, Song et al. (47) showed that miR-146a promoted polarization of M2 macrophages and finally led to increased survival in septic mice. MSC-EVs also contain a substantial quantity of angiopoietin-1 (Ang-1) mRNA, which plays an essential role in vascular stabilization and resolving inflammation. In the current study, Tang et al. (48) demonstrated that the therapeutic benefit of EVs in ALI, and their immunomodulatory properties on macrophages, were partly mediated through their content of Ang-1 mRNA. Furthermore, the transfer of miR-451 and mitochondria from MSC-EVs to the alveolar epithelium and macrophages also contributed to preventing ALI (49). Altogether, these data indicate that MSC-EVs may represent a novel therapeutic option for sepsis-induced ALI.

### MSC-EVs and Sepsis-Induced Actue Kidney Injury

Mounting studies have provided convincing evidence that MSCs exert their renoprotective effects by releasing EVs in several acute kidney injury (AKI) models. AKI is commonly present in more than 20% of septic patients (50). However, data about the therapeutic effects of MSC-EVs in sepsis-induced AKI are scarce. Currently, the anti-apoptosis (51), pro-angiogenesis (52), and anti-inflammatory (53) properties of MSC-EVs have been considered as the important mechanistic approaches to ameliorate AKI induced by different causes. In AKI mice models induced by ischemia-reperfusion injury, Bruno et al. (27) found that MSC-EVs induced the expression of anti-apoptotic genes (Bcl-XL, Bcl2, and BIRC8) in renal tubular epithelial cells while simultaneously down-regulating pro-apoptotic genes (Casp1, Casp8, and LTA), thus reducing tubular cell apoptosis and conferring an anti-apoptotic phenotype necessary for tissue repair. Interestingly, the anti-apoptosis effect of MSC-EVs may be mediated in part by the transfer of miRNAs (miR-223 and miR-199a-3p) to tubular cells (54, 55). In addition, MSC-EVs can directly shuttle several pro-angiogenesis transcription factors including vascular endothelial growth factor (VEGF) (52), insulin-like growth factor 1 (IGF-1) (56), and basic fibroblast growth factor (bFGF) (57) to the damaged renal tubular epithelial cells and enhance angiogenesis, which is considered as an important step in tissue regeneration. In a mouse model of AKI induced by glycerol, Bruno (58) demonstrated that 15 μg of MSC-EVs injected intravenously activated the proliferative progress in viable tubular cells through their pro-angiogenesis effects. Importantly, the effects of MSC-EVs on the recovery of AKI were as effective as their parental MSCs. It is well known that excessive inflammation is the culprit leading to sepsis-induced AKI. MSC-EVs can modulate renal inflammation by reducing the release of several pro-inflammatory cytokines, including IL-1, IL-6, and TNF-α, while restoring the level of the anti-inflammatory cytokine IL-10 (26, 53, 59). This anti-inflammatory effect may be associated with the C-C motif chemokine receptor-2 (CCR2) contained within MSC-EVs, which can suppress macrophages function and attenuate renal injury (60). At present, MSC-EVs have been studied in patients with chronic kidney disease (61). The results showed that administration of MSC-EVs had no side effects and caused significant improvement of the kidney function. Collectively, there are good reasons to believe that MSC-EVs could be exploited as a new therapeutic approach for sepsis-induced AKI.

### MSC-EVs and Sepsis-Associated Cardiovascular Disorder

Despite only a few studies having addressed potential cardioprotective effects of MSCs on sepsis-induced cardiovascular disease, the data still support that MSCs mediate their protection through the secretion of EVs. In septic mice induced by cecal ligation and puncture (CLP), MSC-EVs displayed cardioprotective benefits and enhanced survival of cardiomyocyte cells. In this research, Wang et al. (28) further proved that miR-223 enriched in MSC-EVs was critical for EVs-elicited action in sepsis. miR-223 can inhibit the expression of Sema3A and Stat3, negatively regulating the expression of many inflammatory genes, such as TNF-α, IL-6, and IL-1 (62). Another important piece of research by Tabet et al. (63) also mentioned that miR-223 was able to alleviate the inflammatory process in human coronary artery endothelial cells by downregulating intercellular adhesion molecule 1. In addition, some anti-apoptotic miRNAs contained within MSCs-EVs showed a vital role in cardioprotection. For example, miR-221 can reduce the expression of p53 upregulated modulator of apoptosis and is more highly enriched in MSC-EVs than that in their parent MSCs. Numerous studies (34, 64) have confirmed that the delivery of miR-221 contained within EVs contributed to the cardioprotection by MSC-EVs. A study by Yu et al. (65) demonstrated that MSC-EVs enriched with anti-apoptotic miR-19a also restored cardiac function and reduced infarct size in a rat model of acute myocardial infarction (AMI). In addition to miRNAs, the protein of CD73 on the surface of MSC-EVs was suggested to overcome the pro-apoptotic milieu of the perfused myocardium (66). In AMI mice models, Bian et al. (67) reported that intramyocardial injection of MSC-EVs markedly promoted angiogenesis and reduced infarct size. They further identified that MSC-EVs might be a mixture of miR-150-enriched MVs and Exos (33). Moreover, the pro-angiogenesis effect of the other cargo of MSC-EVs had been investigated *in vitro* and *in vivo* MI models, including miR-210 (66), miR-21 (68), platelet-derived growth factor D (PDGF-D) (69), hypoxia-inducible factor 1-alpha (HIF1-α) (70), and extracellular matrix metalloproteinase inducer (EMMPRIN) (71) and angiopoietin (72). Recently, Xiao et al. (73) demonstrated that the beneficial effects offered by MSCs in MI model were at least partially because of improved autophagic flux through excreted EVs containing mainly miR-125b-5p. On the basis of these studies, MSC-EVs seem to be an ideal therapeutic agent for sepsis-induced cardiovascular disease in the near future.

### MSC-EVs and Sepsis-Induced Liver Injury

During sepsis, liver injury occurs frequently and contributes to the pathogenesis of multiple organ dysfunction, and has been considered as an early indicator of poor outcome in septic patients (74, 75). Studies in a range of liver disease models demonstrated that MSC-EVs could suppress inflammatory response, reduce hepatocyte apoptosis, and enhance liver regeneration. However, few applications of MSC-EVs into sepsis-induced liver injury are available. Nong et al. (76) studied the hepatoprotective effect of MSC-EVs in a mouse model of hepatic ischemia-reperfusion injury. They showed that human-induced pluripotent stem cell-derived MSC-EVs protected hepatocytes via reducing pro-inflammatory cytokine production, such as TNF-α, IL-6, and high mobility group box 1 (HMGB1). Likewise using a concanavalin-A-induced liver injury model, Tamura et al. (77) demonstrated that MSC-EVs decreased production of pro-inflammatory cytokines, while anti-inflammatory cytokine and regulatory T cell levels increased. Recently, the anti-apoptotic and anti-oxidant effects of MSC-EVs were also investigated. A study in a lethal mouse model of liver failure induced by D-galactosamine/TNF-α showed that MSC-EVs containing Y-RNA-1 activated anti-apoptotic pathways, thus mitigating hepatic injury and enhancing survival (78). In carbon tetrachloride (CCl4)-induced liver injury, Yan et al. (79) demonstrated that MSC-EVs promoted the recovery of hepatic oxidant injury through the delivery of glutathione peroxidase 1 (GPX1). Additionally, Tan et al. (80) confirmed that MSC-EVs were capable of promoting hepatocyte regeneration by inducing the IL-6/STAT3 pathway and cell cycle progression. Proteins in MSC-EVs, such as interleukin 6 signal transducer (IL6ST), chemokine Ligand 2 (CXCL2), and hepatocyte growth factor (HGF) etc., were found to be involved in the liver-regeneration process (80). However, it remains to be determined whether miRNAs or other mRNAs within MSC-EVs may have any impact on the proliferation effect of the hepatocytes. According to these studies, MSC-EVs may be considered as a novel therapeutic approach for alleviating sepsis-induced liver injury, and correlative research should fill the gap in this field in the future.

## Advantages and Challenges of MSC-EVS Therapy

Compared to MSC-based therapy, MSC-EVs therapy offers more advantages. First, MSC-EVs are highly stable and suitable for long-term storage without the addition of potentially toxic cryopreservatives (21, 81, 82). Second, MSC-EVs can induce stronger signaling in intercellular communication by directly transferring functional proteins and miRNAs to the recipient cells (83, 84). Third, MSC-EVs have no heterologous risk (85, 86) and no immune response after allogeneic application (87). Furthermore, a significant advantage of MSC-EVs is to circumvent the potential tumorigenicity of MSCs. So far, there is no evidence showing the oncogenic potential of MSC-EVs. A study (88) reported that MSC-EVs can inhibit tumor growth and exert an anti-tumor activity both *in vitro* and *in vivo*. Given this, MSC-EVs look to be a novel and promising therapeutic approach for sepsis.

However, there remain significant challenges that need to be addressed prior to the potential application of MSC-EVs in human sepsis. First, the protocol for isolation has to be standardized. Currently, no “gold standard protocol” for EVs isolation exists, instead different isolation methods are used to isolate different subsets of EVs even though the source is the same. Second, the suitable markers to analyze EVs at a single-vesicle level must be established. So far no consensus exists regarding the nomenclature of EVs or the markers that distinguish each type of EV once they have been secreted or shed from the cells. Third, there is a paucity of knowledge about the content within MSC-EVs. EVs contain a trove of cellular cargos, which are able to act as diverse functions and can vary widely between sources and conditions. It is unknown which effector molecules are important within EVs or which are not protective or harmful. Meanwhile, there is a critical need for exploiting possible ways to modify MSC-EVs composition and modulate their biological activities. Fourth, the therapeutic capacity of MSC-EVs derived from different sources in sepsis should be investigated. In addition, much work is needed on how to optimize the methods to produce MSC-EVs on a large scale, how to validate the dosage and half-life of MSC-EVs, and confirming the potential effects of MSC-EVs in the late stage of sepsis. Finally, the unknown negative effects of MSC-EVs have to be clarified.

## Conclusions

Extracellular vesicles (EVs) are naturally released from almost any cell and participate in cell-to-cell communication in physiological as well as pathological processes by transferring their components, such as proteins, miRNA, mRNA, and even mitochondria. A study (89) considered EVs as possible culprits during the pathogenesis of sepsis, whereas EVs derived from MSCs showed striking therapeutic benefits in sepsis. In this review, we have summarized the recent knowledge related to the therapeutic applications of MSC-EVs in sepsis. These considerable preclinical data support the hypothesis that cell-free therapy with MSC-EVs could be a novel alternative MSC-based therapy in sepsis, especially in early stage sepsis. However, realizing this promising therapeutic approach of MSC-EVs would require extensive testing to validate their safety and efficacy.

## Author Contributions

YC and XC wrote the first draft of this article and designed the figure. LQ critically revised the manuscript for important intellectual content. All authors approved the final version.

## Conflict of Interest

The authors declare that the research was conducted in the absence of any commercial or financial relationships that could be construed as a potential conflict of interest.
